# Exploring Auditory Hallucinations in Congenital Deafness and Subvocal Articulation Hypotheses – a Case Report

**DOI:** 10.1192/j.eurpsy.2025.2055

**Published:** 2025-08-26

**Authors:** R. A. Moreira, M. Marinho, J. Justo

**Affiliations:** 1 Psiquiatria, Unidade Local de Saúde do Nordeste, Bragança; 2 Psiquiatria, Unidade Local de Saúde do Santo António, Porto, Portugal

## Abstract

**Introduction:**

One of the main symptoms of schizophrenia is auditory hallucinations. Although reliable epidemiological data is limited, the prevalence of schizophrenia among the deaf community seems to be about the same as in the general population. Approximately 50% of deaf individuals diagnosed with schizophrenia report ‘hearing’ voices or experiencing the sensation of communication in the absence of external input. Minimal information is available regarding the perceptual aspects of voice hallucinations in congenitally deaf individuals.

**Objectives:**

With this case report, we aim to describe auditory hallucinations in a patient with congenital deafness and discuss auditory hallucinations in deaf individuals. We will also examine how these phenomena may fit into explanatory subvocal articulation hypotheses, providing insights into language modality and auditory deprivation.

**Methods:**

Description of a clinical case of a deaf patient with auditory hallucinations in a psychiatric outpatient consultation.

**Results:**

The authors report a case of a 26-year-old male patient with congenital deafness and no prior psychiatric or relevant medical history. He communicated preferably by sign language and also by lip reading. The consultation was realized with the presence of a sign language interpreter. The patient was referred to a psychiatric outpatient appointment due to a 1-year history of bizarre behavior, social isolation, and apparent psychotic symptoms. He lived with his parents and was currently unemployed. According to his mother and brother, he “spoke and laughed alone, like he was talking with someone”. In his mental status examination, he presented a religious delusion and likely auditory hallucinations. He had no changes in his physical and neurologic examination. Blood tests, urinalysis, and urine screening for drug abuse were performed, with normal results. Computed tomography of the brain and an electroencephalogram were also performed, with no significant changes. Over time, the patient was diagnosed with schizophrenia and was medicated with paliperidone with remission of his symptoms.

**Conclusions:**

These findings highlight how auditory hallucinations in congenitally deaf individuals offer a unique view of the interplay between sensory perception and the experience of ‘voices.’ The subvocal articulation hypothesis suggests that auditory hallucinations may arise from disruptions in inner speech mechanisms—subtle, unconscious movements that the brain misinterprets as external voices. In deaf individuals, this experience may manifest through sensory feedback loops adapted to visual modalities, such as the perception of sign language movements, rather than through purely auditory sensations. This shows how language and sensory experiences shape hallucinations, emphasizing the need to consider sensory differences in psychotic symptoms across populations.

**Disclosure of Interest:**

None Declared

